# Associations between Hospital Setting and Outcomes after Pediatric Appendectomy

**DOI:** 10.3390/children10121908

**Published:** 2023-12-10

**Authors:** Anshul Bhatnagar, Sean Mackman, Kyle J. Van Arendonk, Sam Z. Thalji

**Affiliations:** 1 Baylor College of Medicine, Houston, TX 77030, USA; anshul.bhatnagar@bcm.edu; 2 Department of Emergency Medicine, Medical College of Wisconsin, Milwaukee, WI 53226, USA; smackman@mcw.edu; 3 Division of Pediatric Surgery, Department of Surgery, Medical College of Wisconsin, Milwaukee, WI 53226, USA; 4 Department of Pediatric Surgery, Nationwide Children’s Hospital, Columbus, OH 43205, USA; 5 Department of Surgery, Medical College of Wisconsin, Milwaukee, WI 53226, USA; sthalji@mcw.edu

**Keywords:** appendectomy, hospital setting, care access, patient transfer

## Abstract

Prior studies of associations between hospital location and outcomes for pediatric appendectomy have not adjusted for significant differences in patient and treatment patterns between settings. This was a cross-sectional analysis of pediatric appendectomies in the 2016 Kids’ Inpatient Database (KID). Weighted multiple linear and logistic regression models compared hospital location (urban or rural) and academic status against total admission cost (TAC), length of stay (LOS), and postoperative complications. Patients were stratified by laparoscopic (LA) or open (OA) appendectomy. Among 54,836 patients, 39,454 (73%) were performed at an urban academic center, 11,642 (21%) were performed at an urban non-academic center, and 3740 (7%) were performed at a rural center. LA was utilized for 49,011 (89%) of all 54,386 patients: 36,049 (91%) of 39,454 patients at urban academic hospitals, 10,191 (87%) of 11,642 patients at urban non-academic centers, and 2771 (74%) of 3740 patients at rural centers (*p* < 0.001). On adjusted analysis, urban academic centers were associated with an 18% decreased TAC (95% CI −0.193–−0.165; *p* < 0.001) despite an 11% increased LOS (95% CI 0.087–0.134; *p* < 0.001) compared to rural centers. Urban academic centers were associated with a decreased odds of complication among patients who underwent LA (OR 0.787, 95% CI 0.650–0.952) but not after OA. After adjusting for relevant patient and disease-related factors, urban academic centers were associated with lower costs despite longer lengths of stay compared to rural centers. Urban academic centers utilized LA more frequently and were associated with decreased odds of postoperative complications after LA.

## 1. Introduction

Appendectomy is the most common pediatric surgical procedure with nearly 100,000 operations performed annually in the United States at all types of hospitals [1]. The decision to transfer pediatric patients to urban academic hospitals must be weighed against burdens which may include disruption in continuity of care, higher cost to the family, longer hospitalizations, and increased patient travel distances [2,3,4]. In order to define this balance for different pediatric patients with appendicitis, it would be useful to know more specifically which patient, disease, or treatment factors are associated with the potential benefits of transfer to an urban academic hospital. Furthermore, a recent study reviewing operative costs for laparoscopic appendicitis among both adult and pediatric patients in the United States found significant variability that did not correlate with case volume per site [5]. Given the recent focus on cost stewardship in the literature, it is important to define how admission costs for appendicitis may vary by operative approach and disease complexity as this may elucidate opportunities for cost reduction at all sites.

Hospital characteristics such as urban/rural location and teaching status are associated with differences in outcomes after appendectomy. However, prior studies have not adjusted for significant differences in patient and treatment patterns between settings when comparing outcomes using pooled analysis of all patients regardless of operative approach [6,7,8,9,10,11]. The decision to proceed with a laparoscopic appendectomy (LA) versus an open appendectomy (OA) depends not only on the individual patient and disease characteristics but also on the resources and experience at the treating hospital. The choice of operative approach (or need for intraoperative conversion) often reflects important disease-related characteristics that are not accounted for in prior database-driven analyses. The associations between operative approach, hospital setting, and subsequent outcomes thus remain unclear.

In this study, we examine these associations using a nationally representative database after stratifying by operative approach. Our hypothesis is that the associations between hospital setting and outcomes (including total admission cost, length of stay, and postoperative complications) differ by operative approach and by patient and disease characteristics. These associations may inform clinicians when deciding when to transfer pediatric patients to tertiary centers.

## 2. Materials and Methods

### 2.1. Data Source

All data were retrospectively analyzed from the national Kid’s Inpatient Database (KID), produced by the Healthcare Cost and Utilization Project (HCUP). HCUP-KID is the largest all-payer pediatric (age ≤ 20) inpatient database in the United States and is published every three years. The 2016 HCUP-KID used in this study contains clinical and non-clinical information for approximately three million pediatric hospitalizations. This version includes discharges limited to the 2016 calendar year. The database, when weighted, accounts for nearly seven million pediatric discharges from over four thousand U.S. hospitals. Non-newborn pediatric discharges, such as appendicitis cases, are sampled at a rate of 80%.

### 2.2. Patient Demographics and Disease Classification

Subjects and disease characteristics were identified using relevant International Classification of Diseases, Tenth Revision (ICD-10) codes. Subject inclusion required both an ICD-10 diagnosis code (K35, K36, K37) indicating a primary diagnosis of appendicitis and a procedure code (families 0DTJ- and 0DBJ-) indicating appendectomy. We excluded cases where an appendectomy was performed for a diagnosis other than appendicitis and cases where appendicitis was treated with a non-surgical approach. OA was identified by specific procedure codes 0DTJ0ZZ (Resection of Appendix, Open Approach) and 0DBJ0ZZ (Excision of Appendix, Open Approach), whereas all other procedure codes were classified as LA. Complex (perforated) appendicitis was identified using codes K35.2, K35.20, K35.21 indicating generalized peritonitis with or without abscess [12]. Patient demographics included age, race, ethnicity, and gender, all of which have been shown to influence appendectomy outcomes [6,13,14]. HCUP categorizes race/ethnicity into White, Black, Hispanic, Asian, Native American, and other (for this study, the latter three were grouped as other due to smaller sample sizes). Race/ethnicity was missing in 6% of patients in the database, and these patients were re-coded as “missing” for the purpose of subsequent analysis. All other independent variables were missing in <0.01% of all patients.

### 2.3. Defining Hospital Characteristics

Treating facilities were categorized into three groups: rural hospitals, urban non-academic hospitals, and urban academic hospitals. The 2016 KID defines a teaching hospital as a hospital that either “has one or more Accreditation Council for Graduate Medical Education approved residency programs, is a member of the Council of Teaching Hospitals or has a ratio of full-time equivalent interns and residents to beds of 0.25 or higher” [15]. The distinction between an urban and rural hospital for KID relied on its core based statistical area (CBSA) type, which is determined by the United States Office of Management and Budget. A hospital in a county classified as a CBSA type of “metropolitan” was considered urban, while those in counties with a CBSA type of “micropolitan” or “non-core” were considered rural.

### 2.4. Defining Outcomes

This study had three outcome variables: total admission cost (TAC), length of stay (LOS), and incidence of postoperative complications. Total hospital charges were converted to TAC using the database provided cost-to-charge ratios, as cost is a more standardized measure of financial burden. LOS was defined as the number of days between hospital admission and discharge. Postoperative complications were identified using ICD-10 diagnosis codes for complications including surgical site infections and intraabdominal abscess (T81.4–6) as well as other complications related to surgery that are commonly included in similar analyses [8,9,16] (Table 1).

### 2.5. Statistical Analysis

Medians and interquartile ranges (IQRs) were calculated for continuous variables. Frequencies and percentages were used to classify categorical variables. Continuous variables were compared using the median test for significance. χ^2^ tests were used to analyze categorical variables. Univariable and multivariable linear regression models were used to determine independent relationships between patient factors, hospital setting, TAC, and LOS. Univariable and multivariable logistic regression models were developed to determine relationships between patient factors, hospital setting, and postoperative complications. For these models, TAC and LOS were natural-log-transformed to account for the significant positive skew present in the data (Shapiro–Wilk testing showed that TAC and LOS data were non-normal (*p* < 0.001)). Natural-log-transformed regression coefficients can be approximately interpreted as the percent change in the dependent variable for every 1-unit increase in the independent variable. Models were built using the variables shown in the associated tables, with the addition of LOS included in the models for TAC. Variables with *p*-value of ≤0.05 on univariable analysis were chosen for inclusion within the multivariable models. We then explored the interaction between hospital setting and factors associated with the decision to transfer: patient age, disease complexity, and the use of laparoscopy for the individual patient [17,18,19]. We performed stratified analyses when these interaction terms were significant (*p*-value of ≤0.05) and reported associations between the subgroups.

The sample was weighted using the database-provided weights in order to better represent the national population and obtain national estimates. The Statistical Package for the Social Sciences version 28.0 was used to conduct all statistical analyses. A 2-sided *p*-value of ≤0.05 was considered statistically significant.

## 3. Results

### 3.1. Descriptive Statistics of Patient Demographics across Hospital Setting

In 2016, 54,836 pediatric patients with appendicitis underwent an appendectomy: 39,454 (72%) were performed at an urban academic center, 11,642 (21%) were performed at an urban non-academic center, and 3740 (7%) were performed at a rural center. The median age among all patients was 12 years old (IQR 8–16); urban academic centers treated significantly younger patients than other hospital settings (*p* < 0.001, Table 2). Females comprised 40% of the total cohort; all hospital types treated similar proportions of male and female patients. The overall racial/ethnic background of the cohort was 43% white, 37% Hispanic, and 6% Black. The racial and ethnic compositions at urban hospitals were similar, but rural hospitals treated greater proportions of white patients (*p* < 0.001).

Laparoscopy was utilized for 49,011 (89%) of all 54,836 patients: 36,049 (91%) of 39,454 patients at urban academic hospitals, 10,191 (87%) of 11,642 patients at urban non-academic centers, and 2771 (74%) of 3740 patients at rural centers (*p* < 0.001). Among all 54,386 patients, 16,112 (29%) had complex appendicitis at the time of surgery: 12,783 (32%) of 39,454 patients at urban academic hospitals, 2422 (20%) of 11,642 patients at urban non-academic centers, and 907 (24%) of 3740 patients at rural centers (*p* < 0.001). Rural hospitals treated a higher proportion of patients living within the lowest income quartiles (*p* < 0.001). Of the 3740 patients treated at rural centers, 3443 (92%) patients lived in a rural residence. However, 3070 (47%) of all 6513 patients who lived in rural residences were treated at an urban center.

### 3.2. Unadjusted Outcomes by Disease Complexity, Operative Approach, and Hospital Setting

Patients with complex appendicitis experienced a significantly higher TAC regardless of operative approach or hospital setting (*p* < 0.001, Table 3). Among patients with simple appendicitis, TAC at urban centers was significantly lower than at rural centers for both operative approaches (*p* < 0.001). Patients with complex appendicitis at urban centers experienced longer LOS than at rural centers (*p* < 0.001). The median LOS for simple appendicitis was 2 days regardless of operative approach or hospital setting (*p* < 0.001).

Complications were more common among those with complex disease and after OA. Patients who underwent LA at urban academic centers experienced a lower proportion of complications for both simple (*p* = 0.004) and complex (*p* = 0.003) appendicitis. For patients who underwent OA, there were no significant differences in complications by hospital setting. Patients treated with OA at urban centers experienced significantly lower cost/day than at rural centers (*p* < 0.001).

### 3.3. Adjusted Associations by Disease Complexity, Operative Approach, and Hospital Setting

Multivariable models for factors associated with TAC, LOS, and postoperative complications used covariates that were significant on univariable models (Table 4) including age, race, income, insurance, disease complexity, operative approach, and hospital setting. In adjusted analysis, older age groups were associated with decreased LOS (Table 5); children aged ≥ 13 years were associated with a 32% decreased LOS compared to children aged ≤ 5 years (95% CI −0.344–−0.300). Older age was not associated with a difference in TAC or postoperative complications. Black patients were associated with a 4% increased TAC (95% CI 0.026–0.056), 12% increased LOS (95% CI 0.093–0.144), and 1.4-fold increased odds of postoperative complications (95% CI 1.162–1.618) compared to white patients. Hispanic patients were associated with a 7% increased TAC (95% CI 0.063–0.080) and 1.2-fold increased odds of postoperative complications (95% CI 1.085–1.328) compared to white patients. Patients within higher income quartiles were associated with progressively decreasing LOS and lower odds of postoperative complications; patients within the highest income quartile were associated with a 9% decreased LOS (95% CI −0.110–−0.076) and 0.7-fold decreased odds of complications (95% CI 0.642–0.834) compared to patients within the lowest income quartile.

Patients with complex appendicitis were independently associated with a 17% increased TAC (95% CI 0.161–0.176), 71% increased LOS (95% CI 0.704–0.729), and 2.4-fold increased odds of postoperative complications (95% CI 2.235–2.643) compared to patients with simple appendicitis. Patients who underwent OA were independently associated with a 9% decreased TAC (95% CI −0.103–−0.081), 24% increased LOS (95% CI 0.220–0.257), and 2.2-fold increased odds of postoperative complications (95% CI 1.940–2.396) compared to patients who underwent LA. Urban academic centers were associated with an 18% decreased TAC (95% CI −0.193–−0.165) despite an 11% increased LOS (95% CI 0.087–0.134) compared to rural centers. Among all pooled patients, hospital setting was not associated with a difference in the odds of postoperative complications.

### 3.4. Stratified Analyses by Age, Disease Complexity, and Operative Approach

We carried out stratified analyses based on significant interaction terms between hospital setting and patient age, disease complexity, and operative approach to explore how outcomes may vary within these subgroups. After stratifying by age group, urban academic centers remained associated with decreased TAC and longer LOS within all age groups and there were no significant associations between hospital setting and postoperative complications among any age group. After stratifying by disease complexity, urban academic centers remained associated with decreased TAC and longer LOS among both simple and complex appendicitis; however, there was no association between hospital setting and postoperative complications among patients with complex appendicitis. After stratifying patients by operative approach, urban academic centers were associated with a decreased odds of complication among patients who underwent LA (OR 0.787, 95% CI 0.650–0.952). There was no association between hospital setting and postoperative complications among patients who underwent OA.

## 4. Discussion

In a nationwide comparison of pediatric patients who underwent appendectomy by hospital setting, urban academic centers were associated with decreased TAC despite longer LOS among all patients. While overall analysis found no difference in postoperative complications by hospital setting, we found this association to be modified by operative approach. Urban academic centers were associated with a decreased odds of postoperative complications only among patients who received LA with no significant association after OA. Importantly, the utilization of laparoscopy was significantly higher at urban academic centers compared to rural centers, particularly among patients with complex appendicitis. Outcomes were further associated with established socioeconomic factors including race, ethnicity, and income quartile. Nearly all patients treated at rural centers lived in a rural residence. However, half of all patients from rural residences received their operation at an urban center—indicating some inherent patient or physician selection for referral to tertiary care. The findings suggest that while many patients experience equivalent outcomes in various hospital settings, some potentially recognizable populations exist that may benefit from transfer to a tertiary (urban academic) center. This may include patients for whom a laparoscopic approach may not be feasible at an outlying hospital (based on age or disease characteristics) but who would likely receive LA at a referral center. The findings herein may help guide this decision.

In previous studies on hospital setting and outcomes after pediatric appendectomy, urban centers were associated with lower daily costs, longer LOS, and decreased odds of postoperative complications [6,7,8,9,10,11]. These papers universally examined all pediatric patients with appendicitis in pooled analyses that may be biased by the differences in patient populations and treatment patterns inherent to each setting. For instance, rural centers consistently utilize OA more frequently and urban centers treat a higher proportion of complex appendicitis. After stratifying the patients in the current paper by disease complexity and operative approach, we found that hospital setting was similarly associated with cost and LOS among the subgroups. However, the association between urban academic centers and decreased odds of complications was significant only for patients who underwent LA and not among patients who underwent OA. This may have been masked in previous analyses that pooled operative approach and may be a clinically important consideration. Surgeons recognize that certain factors (perforation, obesity, higher leukocyte counts, periappendicular abscess, or diffuse peritonitis) increase the chance of OA [20,21,22,23]. While urban academic centers address a higher proportion of complex appendicitis laparoscopically, our data suggest that those patients who ultimately undergo OA may experience similar outcomes despite staying closer to home and family.

The factors associated with care at a tertiary children’s hospital are a recent focus in the literature. Georgeades et al. examined pediatric patients with appendicitis in the state of Wisconsin and observed that just 32.2% of children underwent appendectomy at the hospital closest to their home [17]. They further found that care at a major children’s hospital was associated with younger age, Black race, Hispanic ethnicity, complex appendicitis, and patient residence characteristics such as rurality, distance to hospital, and lower area deprivation index. The national sample in the current analysis revealed similar clinicodemographic trends for care at an urban academic center with nearly half of all patients living in rural residences ultimately having surgery at an urban center. Patient selection for transfer or self-referral already occurs in clinical practice, and further work may delineate which patient populations benefit most from care at a tertiary center. The benefits of care at an urban academic center are related resource utilization as well, with a USD 968 lower cost/day after OA and USD 741 lower cost/day after LA. This is in contrast to a recent paper examining outcomes in relation to payment rate to compare the “care value” between children’s hospitals and non-children’s hospitals for thirteen common procedures including appendectomy [24]. They found that outcomes were comparable despite higher costs of care at children’s hospitals, which again highlights the need to determine value at a more granular level. Regardless, potential benefits of transfer must be weighed against the cost to families. These familial costs include transportation, lodging, and interruption to work schedules, and the emotional burden of care far from home [25]. The selective association between care at an urban academic center and decreased odds of postoperative complications among patients who undergo LA may reflect a greater experience in laparoscopy among pediatric patients at urban centers due to higher case volume. The utilization of laparoscopy is significantly higher at urban academic centers (>90% in this report) and remains high even among patients with complex appendicitis [8,11,26,27,28]. Finally, the timing of operative intervention may differ between centers. In a recent retrospective analysis, children who underwent laparoscopic appendectomy at a single academic institution experienced no difference in outcomes when comparing daytime versus nighttime operations [29]. For patients presenting to outlying hospitals where non-emergency cases are more commonly postponed to a morning shift, there may be a benefit to transferring to a tertiary center that can then accommodate immediate intervention, especially among patients with complex appendicitis [30].

Differences in outcomes between hospital settings may also be associated with well-documented socioeconomic disparities. In this cohort, nearly all patients in the highest income quartile were treated at urban centers while rural hospitals treated a greater proportion of patients from the lowest quartile. Rural centers also almost solely treated individuals who lived in rural areas. Patients living in rural areas experience lower household incomes, diminished access to healthcare providers and resources, and more commonly have public insurance or no insurance compared with their counterparts from urban residences [31,32,33]. Furthermore, Black and Hispanic patients were more likely to experience higher costs, longer hospital stays, and increased odds of postoperative complications regardless of operative approach. These are consistent with known racial disparities in healthcare [34,35]. Disadvantages in socioeconomic support outside of the hospital for families of certain races/ethnicities may discourage or prevent patients from seeking early and effective medical treatment. Black and Hispanic patients are also at increased risk of poor access to healthcare, which may lead to delays in care and more advanced disease at presentation [36]. This can lead to poor outcomes for patients, increased healthcare costs, and inefficient hospital utilization that burdens the healthcare system. Efforts that aim to increase geographic and racial/ethnic equity and access to care may lessen these disadvantages.

Our study shares its limitations with other HCUP-KID and database studies. HCUP-KID is a billing database which records diagnoses and procedures through ICD-10 codes; these codes can be missing or misclassified. However, rates of laparoscopic utilization, complex appendicitis, and postoperative complications in this analysis are comparable to studies that define these same factors in other databases, which suggests consensus in the methods used to define these metrics [37]. HCUP-KID does not track important clinical factors like postoperative pain, long-term outcomes, or re-admission rates (re-admission rates following appendectomies are around 3–5%). The database does not discern which patients who ultimately underwent OA had started with a laparoscopic approach. We calculated TAC using the database-provided cost-to-charge ratios, but the results may not fully account for the actual cost of care. The database does not define the proportions of TAC that are associated with the perioperative versus postoperative phases. The relationship between TAC in these findings may be different when examining outcomes in other countries with different payment and reimbursement systems. The database does not contain information on several important socioeconomic factors including driving distance from a patient’s residence to the treating hospital, which is an important factor that can influence hospital choice and subsequent outcomes. Surgeon information, including specialty in pediatric surgery, is not captured by HCUP and cannot be delineated in these data. Surgeon specialty is likely a major driver of the findings of this paper which is incompletely controlled for by stratifying centers into academic versus non-academic. The administrative billing record-based model of HCUP-KID and similar large databases lends itself to bias. The relationship between access to care and outcomes for pediatric appendectomy must be further explored with more granular datasets.

## 5. Conclusions

In conclusion, among pediatric patients with appendicitis, urban academic centers were associated with lower costs despite a longer LOS compared to rural centers. Urban academic centers were associated with decreased odds of postoperative complications after LA. However, there was no difference in the odds of postoperative complications after OA. Urban centers utilized laparoscopy at a significantly higher rate than rural centers despite treating a greater proportion of patients with complex appendicitis. It is not realistic or advisable to recommend that all children undergo appendectomy at an urban academic center, and the results presented herein may help physicians to make informed decisions about when to transfer to a tertiary center.

## Figures and Tables

**Table 1:** ICD-10 Diagnosis Codes for Postoperative Complications.

Category	Subcategory	ICD-10 Code
Respiratory	Pneumonia	J15, J18
	Other respiratory failure	J80, J95, J96
Cardiac	Cardiac arrest, myocardial infarction, acute ischemic heart disease, cardiovascular shock	I21–22, I24, I46, T81.1
Infections	Sepsis	A40, A41
	Surgical site (superficial and deep incisional, intraabdominal/pelvic abscess)	T81.4–6
	Other (Clostridium difficile)	A04.7
Rupture	Surgical wound rupture	T81.3
Nervous system	Stroke	I63
Embolism	Pulmonary embolism, DVT	I26, T81.7
Anaesthesia	Anaesthesia-related complications	T88.2–9
Renal	Acute renal failure	N17
Other	Other unspecified complications relating to a surgical procedure	T88.0

We used the above ICD-10 diagnosis codes to determine whether a patient had a postoperative complication.

**Table 2:** Demographics and Disease Characteristic by Hospital Type.

		Hospital Type			
	Total (n = 54,836)	Urban, Academic (n = 39,454)	Urban, Non-Academic (n = 11,642)	Rural (n = 3740)	*p*-Value
Age in years, median (IQR)	12.0 (8.0)	12.0 (8.0)	14.0 (8.0)	13.0 (7.0)	<0.001
Gender (female), n (%)	22,159 (40.4)	16,026 (40.6)	4683 (40.2)	1450 (38.8)	0.081
Race/Ethnicity, n (%)					<0.001
White	23,279 (42.5)	15,893 (40.3)	4892 (42.0)	2494 (66.7)	
Black	3275 (6.0)	2573 (6.5)	524 (4.5)	178 (4.8)	
Hispanic	19,999 (36.5)	14,494 (36.7)	5015 (43.1)	490 (13.1)	
Other	4959 (9.0)	3806 (9.6)	864 (7.4)	289 (7.7)	
Missing	3324 (6.1)	2688 (6.8)	347 (3.0)	289 (7.7)	
Household income, n (%)					<0.001
1st quartile	16,254 (29.6)	11,423 (29.0)	3116 (26.8)	1715 (45.9)	
2nd quartile	13,322 (24.3)	9184 (23.3)	2785 (23.9)	1353 (36.2)	
3rd quartile	13,161 (24.0)	9466 (24.0)	3234 (27.8)	461 (12.3)	
4th quartile	11,400 (20.8)	8911 (22.6)	2382 (20.5)	107 (2.9)	
Insurance status, n (%)					<0.001
Public	27,217 (49.6)	19,824 (50.2)	5650 (48.5)	1743 (46.6)	
Private	23,428 (42.7)	16,758 (42.5)	5090 (43.7)	1580 (42.2)	
Self-pay	2519 (4.6)	1677 (4.3)	583 (5.0)	259 (6.9)	
Other/Unknown	1608 (2.9)	1155 (2.9)	311 (2.7)	142 (3.8)	
Location of residence, n (%)					<0.001
Non-rural	48,322 (88.1)	36,909 (93.5)	11,116 (95.5)	297 (7.9)	
Rural	6513 (11.9)	2545 (6.5)	525 (4.5)	3443 (92.1)	
Disease status, n (%)					<0.001
Simple	38,724 (70.6)	26,671 (67.6)	9220 (79.2)	2833 (75.8)	
Complex	16,112 (29.4)	12,783 (32.4)	2422 (20.8)	907 (24.2)	
Operative approach, n (%)					<0.001
Laparoscopic	49,011 (89.4)	36,049 (91.4)	10,191 (87.5)	2771 (74.2)	
Open	5825 (10.6)	3405 (8.6)	1451 (12.5)	969 (25.8)	

Table 2 Legend: Patient demographics varied significantly across hospital setting. Compared with rural centers, urban hospitals tended to treat younger, wealthier, and more racially diverse patients. Urban hospitals also more commonly utilized laparoscopy and treated patients with complex appendicitis. Abbreviations: IQR = interquartile range, n = number (frequency count).

**Table 3:** Unadjusted Outcomes by Hospital Type, Stratified by Disease status and Operative Approach.

		Hospital Type			
	Total (n = 54,836)	Urban, Academic (n = 39,454)	Urban, Non-Academic (n = 11,642)	Rural (n = 3740)	*p*-Value
Length of stay in days (LOS), median (IQR)					
Laparoscopic/Simple	2.0 (2.0)	2.0 (2.0)	2.0 (1.0)	2.0 (2.0)	0.031
Laparoscopic/Complex	4.0 (3.0)	4.0 (3.0)	4.0 (3.0)	3.0 (3.0)	<0.001
Open/Simple	2.0 (3.0)	2.0 (3.0)	2.0 (3.0)	2.0 (2.0)	<0.001
Open/Complex	5.0 (4.0)	5.0 (3.0)	5.0 (4.0)	3.0 (3.0)	<0.001
Total admission cost (TAC), median (IQR)					
Laparoscopic/Simple	8292 (4572)	8242 (4692)	8610 (4283)	9423 (5208)	<0.001
Laparoscopic/Complex	11,760 (6868)	12,058 (6998)	11,496 (6700)	11,163 (6150)	0.018
Open/Simple	8058 (5723)	7821 (6378)	7925 (5113)	8883 (5949)	<0.001
Open/Complex	11,821 (9195)	12,122 (10,079)	10,945 (6813)	11,290 (7756)	0.077
Hospitalization cost per day, median (IQR)					
Laparoscopic/Simple	4891 (4019)	4660 (3750)	5384 (4459)	5401 (4954)	<0.001
Laparoscopic/Complex	3022 (1926)	3029 (1823)	3141 (2564)	3302 (3049)	<0.001
Open/Simple	3638 (2109)	3421 (2767)	3443 (2888)	4389 (3682)	<0.001
Open/Complex	2540 (1663)	2452 (1563)	2430 (1514)	3275 (2637)	<0.001
Postoperative complication, n (%)					
Laparoscopic/Simple	1033 (3.0)	681 (2.8)	276 (3.4)	76 (3.6)	0.004
Laparoscopic/Complex	948 (6.6)	727 (6.3)	165 (8.0)	56 (8.2)	0.003
Open/Simple	253 (6.2)	141 (6.3)	75 (6.9)	37 (5.0)	0.238
Open/Complex	245 (14.0)	162 (14.0)	59 (16.2)	24 (10.7)	0.175

Table 3 Legend: Average hospital outcomes following pediatric appendectomy, including length of stay, total admission cost, and postoperative complication rate, varied significantly with hospital setting, operative approach, and disease complexity. Abbreviations: LOS = length of stay, TAC = total admission cost, IQR = interquartile range, n = number (frequency count).

**Table 4:** Univariable Linear and Logistic Regression Models for Outcomes after Appendectomy.

	Cost of Hospitalization			Length of Hospitalization			Postoperative Complications		
Covariate	Coeff	95% CI	*p*	Coeff	95% CI	*p*	OR	95% CI	*p*
Age (continuous)	−0.011	(−0.012, −0.010)	<0.001	−0.038	(−0.039, −0.037)	<0.001	1.005	(0.997, 1.014)	0.23
Age									
≤5 years	Ref	-	-	Ref	-	-	Ref	-	-
6–12 years	−0.181	(−0.198, −0.164)	<0.001	−0.346	(−0.370, −0.322)	<0.001	0.535	(0.469, 0.611)	<0.001
≥13 years	−0.218	(−0.235, −0.202)	<0.001	−0.575	(−0.599, −0.551)	<0.001	0.682	(0.599, 0.775)	<0.001
Gender									
Male	Ref	-	-	Ref	-	-	Ref	-	-
Female	0.004	(−0.005, 0.013)	0.448	−0.011	(−0.025, 0.002)	0.099	0.964	(0.887, 1.046)	0.378
Race									
White	Ref	-	-	Ref	-	-	Ref	-	-
Black	0.083	(0.064, 0.103)	<0.001	0.168	(0.139, 0.186)	<0.001	1.543	(1.316, 1.808)	<0.001
Hispanic	0.071	(0.061, 0.081)	<0.001	0.074	(0.060, 0.089)	<0.001	1.285	(1.173, 1.408)	<0.001
Other	0.040	(0.024, 0.056)	<0.001	0.027	(0.003, 0.051)	0.028	1.248	(1.079, 1.442)	0.003
Missing	−0.020	(−0.039, −0.001)	0.036	0.103	(0.074, 0.132)	<0.001	0.844	(0.691, 1.031)	0.096
Household income									
1st quartile	Ref	-	-	Ref	-	-	Ref	-	-
2nd quartile	0.011	(−0.001, 0.023)	0.082	−0.050	(−0.068, −0.033)	<0.001	0.892	(0.804, 0.990)	<0.001
3rd quartile	0.016	(0.004, 0.028)	0.013	−0.082	(−0.100, −0.064)	<0.001	0.760	(0.681, 0.847)	<0.001
4th quartile	0.022	(0.009, 0.034)	<0.001	−0.150	(−0.017, −0.131)	<0.001	0.618	(0.547, 0.697)	<0.001
Insurance status									
Public	Ref	-	-	Ref	-	-	Ref	-	-
Private	−0.055	(−0.064, −0.046)	<0.001	−0.107	(−0.120, −0.093)	<0.001	0.772	(0.709, 0.841)	<0.001
Self-pay	−0.078	(−0.100, −0.057)	<0.001	−0.119	(−0.151, −0.087)	<0.001	0.868	(0.712, 1.058)	0.161
Other/Unknown	−0.078	(−0.105, −0.052)	<0.001	−0.082	(−0.122, −0.043)	<0.001	0.783	(0.607, 1.011)	0.061
Location of residence									
Non-rural	Ref	-	-	Ref	-	-	Ref	-	-
Rural	0.003	(−0.011, 0.016)	0.654	0.029	(−0.009, 0.049)	0.005	1.305	(1.164, 1.462)	<0.001
Hospital type									
Rural	Ref	-	-	Ref	-	-	Ref	-	-
Urban non-teaching	−0.065	(−0.084, −0.045)	<0.001	−0.036	(−0.065, −0.008)	0.012	0.960	(0.812, 1.135)	0.636
Urban teaching	−0.045	(−0.063, −0.027)	<0.001	0.146	(0.120, 0.172)	<0.001	0.838	(0.719, 0.977)	0.024
Disease status									
Simple	Ref	-	-	Ref	-	-	Ref	-	-
Complex	0.374	(0.365, 0.383)	<0.001	0.766	(0.754, 0.779)	<0.001	2.329	(2.148, 2.525)	<0.001
Operative approach									
Laparoscopic	Ref	-	-	Ref	-	-	Ref	-	-
Open	0.031	(0.017, 0.046)	<0.001	0.254	(0.233, 0.275)	<0.001	2.211	(1.997, 2.449)	<0.001
Disease status and operative approach									
Laparoscopic/Simple	Ref	-	-	Ref	-	-	Ref	-	-
Laparoscopic/Complex	0.370	(0.360, 0.380)	<0.001	0.773	(0.760, 0.786)	<0.001	2.301	(2.102, 2.519)	<0.001
Open/Simple	0.017	(0.001, 0.034)	0.035	0.273	(0.250, 0.295)	<0.001	2.160	(1.875, 2.488)	<0.001
Open/Complex	0.423	(0.399, 0.447)	<0.001	0.987	(0.954, 1.019)	<0.001	5.324	(4.589, 6.176)	<0.001

Table 4 Legend: Multivariable models for factors associated with TAC, LOS, and postoperative complications used covariates that were significant on univariable models including age, race, income, insurance, disease complexity, operative approach, and hospital setting.

**Table 5:** Multivariable Linear and Logistic Regression Models for Outcomes after Appendectomy.

	Cost of Hospitalization			Length of Hospitalization			Postoperative Complications		
Covariate	Coeff	95% CI	*p*	Coeff	95% CI	*p*	OR	95% CI	*p*
Age									
≤5 years	Ref	-	-	Ref	-	-	Ref	-	-
6–12 years	−0.033	(−0.045, −0.020)	<0.001	−0.199	(−0.220, −0.177)	<0.001	0.662	(0.578, 0.757)	<0.001
≥13 years	0.009	(−0.003, 0.022)	0.169	−0.323	(−0.344, −0.300)	<0.001	0.987	(0.861, 1.130)	0.849
Race									
White	Ref	-	-	Ref	-	-	Ref	-	-
Black	0.041	(0.026, 0.056)	<0.001	0.119	(0.093, 0.144)	<0.001	1.372	(1.162, 1.618)	<0.001
Hispanic	0.072	(0.063, 0.080)	<0.001	0.008	(−0.006, 0.021)	0.275	1.201	(1.085, 1.328)	<0.001
Other	0.034	(0.021, 0.046)	<0.001	−0.001	(−0.022, 0.019)	0.889	1.249	(1.077, 1.448)	0.003
Missing	−0.048	(−0.062, −0.033)	<0.001	0.013	(−0.011, 0.038)	0.303	0.844	(0.689, 1.032)	0.099
Household Income									
1st quartile	Ref	-	-	Ref	-	-	Ref	-	-
2nd quartile	0.042	(0.033, 0.051)	<0.001	−0.038	(−0.053, −0.022)	<0.001	0.931	(0.837, 1.034)	0.183
3rd quartile	0.070	(0.060, 0.079)	<0.001	−0.053	(−0.069, −0.037)	<0.001	0.836	(0.746, 0.936)	0.002
4th quartile	0.111	(0.100, 0.120)	<0.001	−0.093	(−0.110, −0.076)	<0.001	0.732	(0.642, 0.834)	<0.001
Insurance status									
Public	Ref	-	-	Ref	-	-	Ref	-	-
Private	−0.022	(−0.029, −0.014)	<0.001	−0.032	(−0.045, −0.018)	<0.001	0.902	(0.820, 0.991)	0.033
Self-pay	−0.041	(−0.057, −0.024)	<0.001	−0.049	(−0.076, −0.020)	0.001	0.862	(0.705, 1.053)	0.147
Other/Unknown	−0.069	(−0.088, −0.048)	<0.001	−0.007	(−0.041, 0.027)	0.692	0.878	(0.678, 1.137)	0.326
Hospital type									
Rural	Ref	-	-	Ref	-	-	Ref	-	-
Urban non-teaching	−0.126	(−0.140, −0.11)	<0.001	0.042	(0.015, 0.067)	0.002	1.097	(0.920, 1.307)	0.301
Urban teaching	−0.179	(−0.193, −0.165)	<0.001	0.111	(0.087, 0.134)	<0.001	0.917	(0.780, 1.078)	0.296
Disease status									
Simple	Ref	-	-	Ref	-	-	Ref	-	-
Complex	0.169	(0.161, 0.176)	<0.001	0.717	(0.704, 0.729)	<0.001	2.431	(2.235, 2.643)	<0.001
Operative approach									
Laparoscopic	Ref	-	-	Ref	-	-	Ref	-	-
Open	−0.093	(−0.103, −0.081)	<0.001	0.239	(0.220, 0.257)	<0.001	2.157	(1.940, 2.396)	<0.001

Table 5 Legend: Regardless of disease complexity, patient demographics, and operative approach, urban hospitals were associated with lower hospitalization costs, despite longer hospitalizations, compared with rural centers. Among all patients, there was no association between hospital setting and postoperative complication rate.

## Data Availability

Restrictions apply to the availability of these data. Data was obtained from HCUP-KID and are available [at URL] with the permission of HCUP-KID. https://hcup-us.ahrq.gov/db/nation/kid/kiddbdocumentation.jsp; Last date of access: 22 December 2022.
